# Arterial Responses to Acute Low-Level Ergot Exposure in Hereford Cows

**DOI:** 10.3389/fvets.2018.00240

**Published:** 2018-10-16

**Authors:** Vanessa Elizabeth Cowan, Alex Neumann, John McKinnon, Barry Raymond Blakley, Taylor Jayne Grusie, Jaswant Singh

**Affiliations:** ^1^Veterinary Biomedical Sciences, Western College of Veterinary Medicine, University of Saskatchewan, Saskatoon, SK, Canada; ^2^Toxicology Centre, University of Saskatchewan, Saskatoon, SK, Canada; ^3^Animal and Poultry Science, College of Agriculture and Bioresources, University of Saskatchewan, Saskatoon, SK, Canada

**Keywords:** caudal artery, *Claviceps purpurea*, cow, ergot, internal iliac artery, median sacral artery, peripheral blood flow, vasoconstriction

## Abstract

Ergot alkaloids are toxic secondary metabolites produced by the fungus *Claviceps purpurea* that contaminate cereal grains. Current Canadian standards allow 2 to 3 parts per million of ergot alkaloids in animal feed. The purpose of this study was to determine whether hemodynamic parameters were altered when beef cows were fed permissible levels of ergot alkaloids (i.e., <3 ppm) on a short-term basis. A dose-response relationship between ergot alkaloid concentration and hemodynamic changes in caudal (coccygeal), median sacral, and internal iliac arteries was hypothesized. Beef cows were randomly allocated to: Control (<15 μg total ergot alkaloids/kg dry matter), Low (132 μg/kg), Medium (529 μg/kg), and High (2115 μg/kg) groups (*n* = 4 per group). Animals were fed 8.8 kg of dry matter daily for 4 days (pre-treatment), 7 days (treatment), and 4 days (post-treatment). The caudal, median sacral, and internal iliac arteries were examined daily using ultrasonography in B-mode and Doppler (color and spectral) mode and hemodynamics endpoints were analyzed by repeated measures mixed model analyses. Caudal artery diameter decreased in the Medium (*p* = 0.004) and High (*p* < 0.001) groups compared to pre-treatment values and the pulsatility index increased (*p* ≤ 0.033) in all ergot treatments during the post-exposure period compared to the Control group. Blood volume per pulse (mL) and blood flow (mL/min) through the caudal artery during the treatment period were reduced in the Medium (−1.0 mL reduction; *p* ≤ 0.004) and High (−1.1 mL *p* ≤ 0.006) groups compared to pre-treatment values. The median sacral artery diameter decreased in the Medium (*p* = 0.006) and High (*p* = 0.017) treatments compared to the Control group. No differences were detected in any hemodynamic endpoints for the internal iliac artery except changes in pulse rate (*p* = 0.011). There was no treatment (*p* > 0.554) or Treatment^*^Time interaction (*p* > 0.471) for plasma prolactin concentration or body temperature. In conclusion, alterations in caudal artery hemodynamics were detected when cows were fed 529 and 2115 μg ergot alkaloids per kg dry matter per day for 1 week. The caudal artery was more sensitive to ergot alkaloids than the median sacral and internal iliac arteries. Our results partially support the hypothesis of a dose-response effect of ergot alkaloids in feed on hemodynamics.

## Introduction

Ergot alkaloids are biologically-active secondary metabolites produced by the pathogenic plant fungus *Claviceps purpurea*. The name “ergot” refers to the dark “sclerotia” produced by the fungus that replace the kernels in the ripe ear of the cereals such as rye, triticale, barley, and wheat. *C. purpurea* infection has become widespread across Western Canada within the past decade (1) and ergot alkaloids are commonly encountered in Canadian and global livestock feeds (2). Exposure to livestock may occur through consumption of contaminated native pasture during grazing or result from feed formulated with contaminated cereal grains and forage.

Ergot toxicity in livestock can manifest in many forms, the most commonly observed of which include the gangrenous, reproductive, and hyperthermic forms. The resulting symptoms are due to the structural similarity of the ergopeptine alkaloids (including ergotamine, ergosine, ergocornine, α-ergocryptine, and ergocristine) to endogenous biogenic amines (i.e., dopamine, serotonin, and norepinephrine) (3, 4). Ergot alkaloids may act as agonists and partial-agonists (4, 5) at these bioamine receptors. A major mechanism of ergotism results from enhanced peripheral vascular constriction by adrenergic receptor blockade and agonism at peripheral serotonin (5-hydroxytryptamine) receptors (4, 6–12). This is implicated in sustained constriction of peripheral arteries that progress to the development of necrosis and gangrene (6, 13). Clinical manifestations of gangrenous ergotism is livestock first appear as hindlimb lameness and swelling of the hooves at the pastern and fetlock regions that progress to necrotic lesions and appendage loss (13–16). Development of gangrenous lesions of the tail tips of cattle has been observed at concentrations as low as 473 μg total ergot alkaloids per kg total ration, while complete hoof loss has been reported at concentrations of 12 000 μg/kg (16), suggestive of a dose-response relationship between ergot alkaloids and gangrenous ergotism.

Fescue toxicosis is caused by the consumption of endophyte (*Acremonium coenophialum*)-infected tall fescue (17–28). Animals experiencing fescue toxicosis also exhibit gangrenous signs similar to ergot poisoning [(29, 30)]. Although related phylogenetically to classic “ergot of rye” (i.e., *C. purpurea*) (31, 32), the ergot alkaloid profiles produced by the endophyte are different than that of *C. purpurea* and, thus, direct comparisons have limited relevance. It is estimated that economic losses to the US livestock industry as a result of tall fescue contamination are ~$1 billion annually (33, 34). Disconcertingly, it is currently unknown how much productivity or performance losses occur to the beef cattle operations due to subclinical *C. purpurea* ergot toxicosis. Furthermore, concentration thresholds for these changes have not been identified in cattle or other livestock species.

Globally, ergot regulations for livestock feed tend to vary. In the European Union, <100 μg/kg (i.e., <0.1% ergot sclerotia by weight) is considered the limit in cereals for livestock consumption. This value is an order of magnitude less in the United Kingdom, i.e., <1 μg/kg (35). In the United States, grain is considered contaminated when sclerotia comprise 0.05–0.3% of grain on a weight basis, depending on the substrate (36, 37). This corresponds approximately to a total ergot alkaloid concentration of 300 μg/kg (35). The current Canadian recommended tolerance level for ergot consumption in cattle is 2,000–3,000 μg/kg of feed (38), which is considerably higher than other countries with ergot regulations for livestock feed.

The purpose of this study was to investigate changes in arterial response in cows associated with increasing ergot alkaloid exposure near the current Canadian tolerance concentration. We hypothesized that low-levels of ergot alkaloids will alter the hemodynamic endpoints of caudal, median sacral and internal iliac arteries in a dose-dependent manner.

## Materials and methods

### Statement of animal ethics

This study was carried out in accordance with the recommendations of the University of Saskatchewan University Committee on Animal Care and Supply (UCACS) and Animal Research Ethics Board (AREB). The protocol was approved by the University of Saskatchewan Animal Care Committee prior to commencing any animal work. Animals were monitored throughout the study for health and wellbeing.

### Ergot alkaloid extraction and quantification procedure with liquid chromatography mass spectrometry (LC/MS)

Quantification of ergot alkaloids in feed with LC/MS was performed as described by Grusie et al. (39). Briefly, all calibrations and analyses were conducted with a high-performance liquid chromatography (HPLC) system (Agilent 1100) fitted with a Agilent Zorbax Eclipse XDB-C18 narrow bore (2.1 × 150 mm; 5 μm) HPLC column that was used in tandem with a mass spectrometer (Micromass Quattro Ultima). The 85/15 (% v/v) extraction solution was prepared using pre-filtered acetonitrile (HPLC grade; EMD Millipore) and 10 mM ammonium acetate (771 mg ammonium acetate in 1 L of fresh barnstead water with a resistance of 16.8 MΩ or higher). Ammonium acetate and acetonitrile solutions were also used as the “Mobile Phase A” and “Mobile Phase B” solutions, respectively. A six-point calibration (i.e., standard) curve was generated by serial dilution of each of the working alkaloid solutions of 1 μg/mL (Romer Labs Inc., Newark, DE, USA) in extraction solution to obtain 12.5, 7.5, 2.5, 1.25, and 0.75 ng/mL. The alkaloids evaluated were ergotamine, ergometine, ergocornine, ergocristine, ergocryptine, and ergosine. Standards were accepted if the standard deviation (i.e., area under the chromatograph curve) did not exceed 15%.

Ground grain and pellet samples (5 g) were diluted in 25 mL of the 85/15 extraction solution and mixed on a magnetic stir plate for 10 min. Supernatants were filtered through a Whatman 41 filter paper into clean glass beakers. In individual glass test tubes, 50 mg of Bondesil-PSA bulk sorbent (40 μm particle size; Agilent Technologies, Santa Clara, CA, USA) was weighed and 400 μL of supernatant was added. Samples were agitated for 45 s and allowed to settle. Supernatant (100 μL) was collected without disturbing the Bondesil-PSA, placed in Agilent auto-sampler vials, a sample volume of 20 μL was injected, and alkaloid detection was performed for 21 min run-times. Individual and total alkaloid concentrations were reported in units of micrograms per kilogram (μg/kg) for each sample.

### Feed formulation and treatment groups

Diets were formulated by diluting highly ergotized pellets with ergot-free pellets, silage, and chopped barley. The ergot-concentrated pellets contained 46,520 μg total ergot alkaloids per kg (Ergocristine: 18229, Ergotamine:12000, α-Ergocryptine: 6965, Ergocornine: 3394, Ergometrine: 3231, and Ergosine: 2,700 μg/kg). The concentrations of ergot in the experimental diet were selected based on the current Canadian guideline of 2,000 to 3,000 μg/kg total ration. Four treatments were employed in this study: negative control, low ergot concentration, medium ergot concentration, and high ergot concentration. Of the 4 kg of pellets fed daily, animals in the control, low, medium, and high treatment groups received 0, 0.025, 0.1, and 0.4 kg of the ergot-concentrated pellets, respectively. Concentration and dose data are provided in Table 1. The weights of pellets included in the ration corresponded to 0, 70, 280, and 1,121 μg total ergot alkaloids per kg ration as fed. On a dry matter basis, these concentrations were 0, 132, 529, and 2115 μg/kg of feed.

**Table 1:** Treatment ration composition and corresponding total ergot alkaloid concentrations of diets fed to lactating Hereford cows.

**Ergot treatment**	**Amount of ergot concentrated pellets (kg)**	**Amount of total ergot alkaloids (μg)**	**Feed dose (μg/kg dry matter)**	**Body weight dose (μg consumed /kg BW)**
Control	0	0	0	0
Low	0.025	1163	132	0.12
Medium	0.1	4653	529	0.58
High	0.4	18610	2115	2.43

*Cows were fed the total mixed ration daily for 1 week during the treatment period. Body weight dosage was calculated for each ergot treatment group using the average weight during the treatment period (see Table 2)*.

Animals were fed at 1.5% of their body weight during the pre-treatment, treatment and post-treatment periods (please see next section for details), corresponding to 16.6 kg of feed (as fed; 4 kg pellets, 11 kg barley silage, 1.6 kg chopped barley per head) per day. This was ~8.8 kg of dry matter. The animals were fed three times daily. The ergotized portion of the ration was fed once daily (during the treatment period of 7 days) at ~0800 h; all cows were fed equivalent amount of ergot-free pellets at this time during the pre- (4 days) and post-treatment (3 days) periods. The cows were fed a mixture of ergot-free pellets, barley, and silage for the remaining two feedings, at ~1000 and 1400 h. Silage used in the total mixed ration contained <15 μg total ergot alkaloids/kg on an as fed basis and <42 μg total ergot alkaloids/kg dry matter. Barley used in the total mixed ration contained < 7.0 μg total ergot alkaloids/kg on an as fed basis and <7.0 μg total ergot alkaloids/kg dry matter.

### Animal husbandry and experimental design

Lactating Hereford cross beef cows (*n* = 16) and their calves were maintained at the University of Saskatchewan Goodale Research Farm. Cows were 48 ± 3 days (mean±SEM) post-partum and were 522 ± 22 kg of body weight at the start of the experiment. The experiment was conducted in the months of June and July. Ambient temperature ranged from 1 to 25.4°C in June and 6 to 28.4°C in July (Saskatoon data from the Environment Canada monthly climate summary). The study was conducted in two replicates (*n* = 8 cows per replicate) 15 days apart to mitigate space and time constraints. Individual cow-calf pair were housed in pens inside the barn (straw bedding, open air access) starting 4 days prior to the feeding of the experimental diet to allow cows to become accustomed to the pelleted ration and the surroundings. Indoor barn temperature and outside ambient temperatures were recorded at the time of ultrasound examination. Animals had *ad libitum* access to water and were monitored daily for general health and for signs of lameness. Rectal temperature was recorded daily between 0900 and 1100 h.

This study included three experimental periods: pre-treatment, treatment, and post-treatment. The pre-treatment period took place for 4 consecutive days (i.e., days−4,−3,−2, and−1). The treatment period occurred for 7 days (i.e., first day of ergot feeding = day 0) immediately following the pre-treatment period. The post-treatment period incorporated 3 days immediately following the treatment period (i.e., days 7, 8, and 9) and 1 day a week later (i.e., day 14).

#### Plasma samples

Blood was collected via jugular venipuncture daily between 0900 and 1100 h using heparinized collection tubes. Plasma was separated from whole blood by centrifugation. Blood samples were stored at −20°C until ELISA analysis for prolactin.

#### B-mode and doppler vascular ultrasonography

Ultrasonography was used to image the caudal, internal iliac, and median sacral arteries on each cow daily. The caudal (coccygeal) artery was selected due to its anatomical location (i.e., the tail) and its examination in similar studies with ergot alkaloids (19, 25). The median sacral artery was selected as the internal counterpart of the caudal artery and likely to be affected less by the changes in ambient temperature due to its location. The internal iliac artery was selected because it is a large elastic artery inside the pelvic cavity that can be reliably imaged at the chosen location over time. Caudal artery was imaged using the transcutaneous approach while the median sacral and internal iliac arteries were imaged using the transrectal approach.

**Figure 1:**
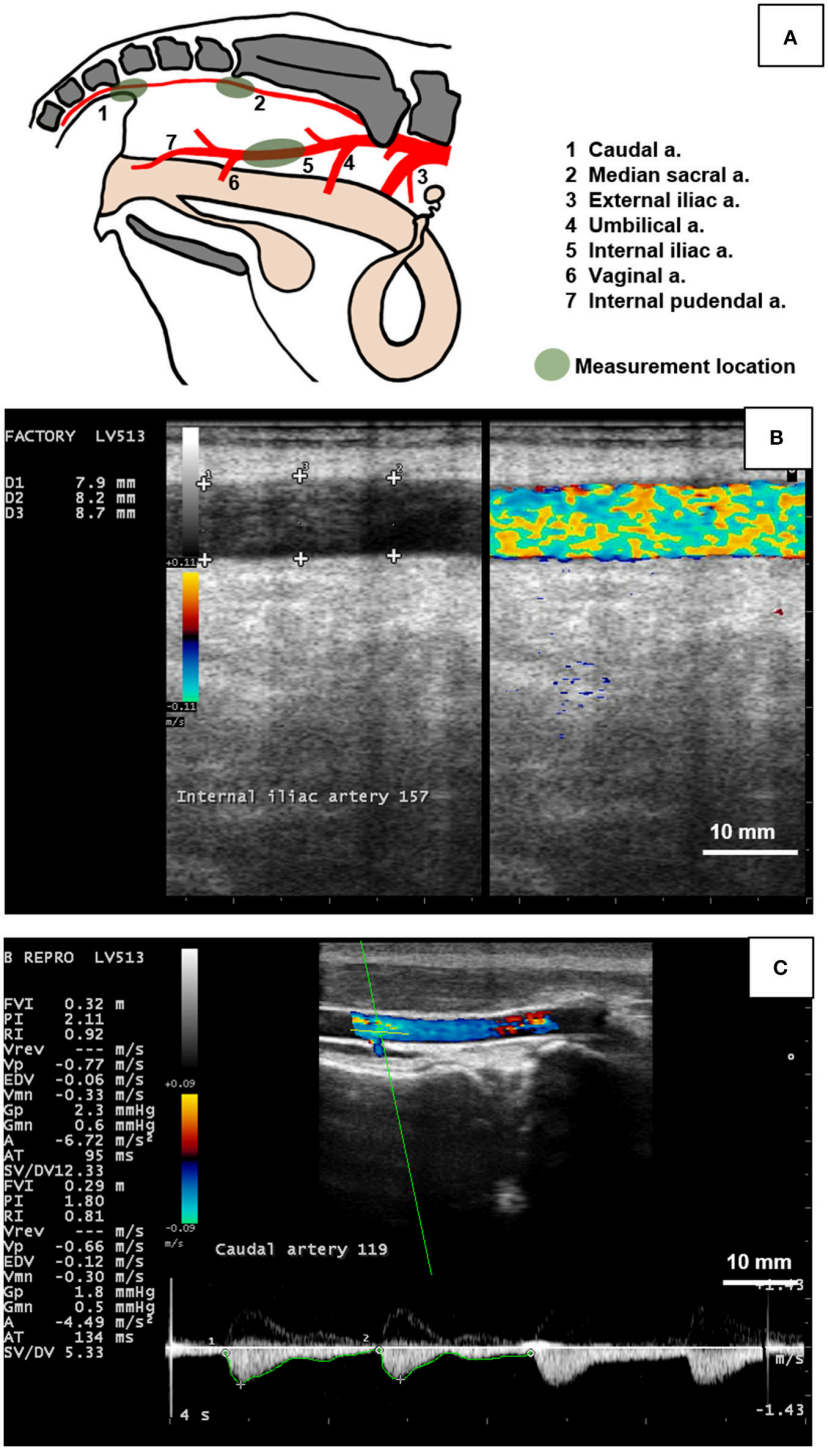
Measurement of the hemodynamic endpoints using B-mode and color Doppler ultrasonography of the caudal, median sacral, and internal iliac arteries in cows. **(A)** Diagram indicating branching pattern of major arteries (1 to 7) in the pelvic cavity and arterial measurement locations (green shaded areas). The caudal artery (1) was imaged transcutaneously at the fourth caudal vertebra. The median sacral artery (2) was imaged transrectally caudal to the sacral ridge at the highest point. The internal iliac artery (5) was measured transrectally between the umbilical (4) and vaginal (6) branches. **(B)** Duplex B-mode and Color Flow Doppler mode of the bovine internal iliac artery in longitudinal section was used to measure arterial diameter at 3 locations (+ signs) to obtain a single (average value) for each artery per day. **(C)** Doppler spectrum waveform (bottom part of image) of the bovine caudal artery was recorded by placing the sampling gate (horizontal and oblique green lines in artery lumen) and used to manually trace the waveform (green line along the bottom of the waveform) to obtain hemodynamic parameters including mean arterial velocity (MnV), peak systolic velocity (PSV), end diastolic velocity (EDV), pulsatility index (PI), and resistivity index (RI). Figure **(A)** was drawn based on Nickel et al. (40). Scale bar for Figures **(B,C)** = 10 mm.

The MyLab™Five ultrasound system (Esaote S.p.A.) with a 7.5 MHz linear-array transducer for transcutaneous and transrectal use. Cows were restrained in a locking head gate prior to ultrasound examination. The caudal artery was imaged transcutaneously at the fourth coccygeal vertebra. The right internal iliac artery was imaged inside the body cavity cranial to the vaginal artery branchpoint. The median sacral artery was measured internally caudal to the sacral ridge at its highest point. Figure 1 depicts the anatomy of the pelvis and measurement locations of the arteries in the present study, as well as ultrasound images captured for hemodynamic variable measurement. The ultrasound transducer was placed on each artery lengthwise to capture a longitudinal section. Videos and images were recorded once a clear image and steady position were achieved. The first set of measurements for each artery was taken in a duplex view of B-mode (brightness mode) and CFM (color flow mode). A 10 s video (audio video interleave (avi) format) was recorded of each artery in the longitudinal aspect to image blood flow and maximum arterial diameter. The second set of measurements were taken in spectral Doppler mode. Spectral waveforms of each artery (minimum three consecutive waveforms) were captured as images. For all arteries, B-mode gain and power were set at 64 and 100%, respectively. Gain for CFM was set to 64% for the caudal artery and 64% for the median sacral and internal iliac arteries. For Spectral Doppler, arteries were positioned at a depth of 5 cm and Doppler angle, spectral gain, and spectral velocity were set to +75°, 58, and 95%, respectively and the sample volume gate was set to 1, 4, and 2 for the caudal, internal iliac, and median sacral arteries, respectively.

### Hemodynamic variable measurements

Images of Doppler spectra were recorded on each sampling day and saved for later analysis. A minimum of three consecutive waveforms were recorded and analyzed for each time point. The video and image files (Esoate's proprietary format) were imported into the MyLabDesk software program (Esaote S.p.A.) for analysis. Video recordings of each artery were taken. For analysis, a longitudinal frozen image of the artery was captured at its maximum diameter. The diameter of each artery was measured using the built-in “distance” function of the software by placing calipers at each lumen boundary (perpendicular to the lumen). Three diameter measurements were taken. Measurements of Doppler spectra were completed using the built-in software functions. Manual tracing of each waveform in the software's built-in “vascular FVI mode” produced a value for peak systolic velocity (PSV; m/s), end diastolic velocity (EDV; m/s), and mean velocity (MnV; m/s), pulsatility index (PI; unitless), and resistivity index (RI; unitless). Pulsatility index is calculated by Systolic Velocity-Diastolic Velocity/Mean Velocity. Resistivity index is calculated by Systolic Velocity-Diastolic Velocity/Systolic Velocity (41). PI and RI are measures of vascular resistance (42), with increased values indicating increased resistance to blood flow (43). The pulse rate was measured using the software's “HR” function by placing measurement calipers at the peak of two consecutive waveforms (units of bpm). Average values from the three consecutive waveforms of each endpoint for each day of the experiment were calculated and evaluated statistically.

Arterial radius, blood volume per beat, and blood flow per minute were derived using the averages of the above variables in the equations as follows:

$$\begin{array}{l} \text{ Radius }(\text{mm});\;R\;=\text{ diameter}/2\\ \text{Blood volume per pulse }(\text{mL});\;VPP\;=\;(\text{Mean arterial velocity}*100)\\ \;(\pi \left(\frac{\text{R}}{100}\right))(\frac{\text{pulse duration}}{1000})\\ \text{ Flow per minute }(\text{mL}/\min);\;FPM\;=\text{ Blood volume per pulse}\\ \;*\text{Pulse Rate} \end{array}$$

### Enzyme-linked immunosorbent assay (ELISA) for bovine prolactin (PRL) (antigen detection)

A commercial competitive inhibition ELISA Kit (44) for Prolactin (PRL) was purchased from CedarLane (Product number CEA846BO). The assay uses biotin-labeled antibody specific for PRL and the company-reported detection range of the kit is 2.47–200 ng/mL (sensitivity < 0.98 ng/mL). Standards (0–200 ng/mL) were run on each plate. All samples were assayed in duplicate on the same day as per kit instructions using the provided reagents. Holstein calf serum (previously determined by the laboratory to have high prolactin concentration) was used as reference standards (undiluted and 1:2 dilution). Inter-assay and intra-assay coefficients of variance were 14.1% (*n* = 6) and 5.1% (*n* = 24), respectively.

### Statistical analysis—repeated measures analysis of variance

Statistical Analysis Software (SAS) version 9.4 with Enterprise Guide 6.1 (SAS Institute, Cary NC USA) was used for all analyses. The repeated measures Mixed procedure was used to test for the effect of treatment (i.e., four ergot concentrations), experimental period (i.e., pre-treatment, treatment, post-treatment), and interactions. (see Supplementary section for the complete syntax). Variables analyzed included body weight, rectal temperature, prolactin concentration, and hemodynamic endpoints (both measured and calculated). Day of data collection was included as a repeated variable and animals were nested within treatment groups. Experimental replicate was included in the model as a random factor. The best fit model for the data was selected based on the smallest Akaike information criteria (AICc) value from the nine tested covariance structures (simple, compound symmetry, heterogeneous compound symmetry, Toeplitz, banded Toeplitz, Huynh-Feldt, autoregressive, heterogeneous autoregressive, and ante-dependence). Final analysis of the data (Type 3 Test of Fixed Effects) included least square means for main effects or interaction terms. Statistical significance was α = 0.05. Multiple comparisons were conducted where applicable using the differences of least square means.

## Results

Average ambient temperatures during the pre-treatment, treatment, and post-treatment periods were 17.9 ± 0.3, 17.2 ± 0.2, and 19.6 ± 0.5°C, respectively. No symptoms of lameness or hyperthermia were observed in any of the animals throughout the duration of the study.

### Plasma prolactin concentration, weight, and rectal temperature

The data for plasma prolactin concentration, body weight, and rectal temperature are provided in Table 2. There was no Treatment (*p* = 0.554) or Treatment^*^Experimental Period interaction (*p* = 0.471) for plasma prolactin concentration. Prolactin concentration varied by Experimental Period (*p* < 0.001). Plasma prolactin (averaged across all groups) decreased (*p* < 0.001) from the pre-treatment period (34.7 ± 1.2 ng/mL) to treatment period (31.0 ± 1.0 ng/mL) followed by a further decrease to the post-treatment period (27.3 ± 1.2 ng/mL).

**Table 2:** Plasma prolactin concentration, body weight, and rectal temperatures (mean±SEM) of lactating Hereford cows (*n* = 4 per treatment group) during the pre-treatment (4 days), treatment (7 days), and post-treatment (4 days) experimental periods to increasing concentrations of ergot alkaloids in their feed in Control, Low, Medium, and High groups.

**Experimental period**	**Ergot treatment**			
	**Control**	**Low 132 μg/kg DM**	**Medium (529 μg/kg DM)**	**High (2115 μg/kg DM)**
**PROLACTIN (ng/ml)**				
Pre-treatment	37.8 ± 2.1	35.6 ± 3.3	31.3 ± 2.2	34.3 ± 1.3
Treatment	35.0 ± 2.6	33.7 ± 1.9	25.6 ± 1.5	29.7 ± 1.7
Post-treatment	30.7 ± 3.4	28.8 ± 2.5	24.4 ± 1.5	25.0 ± 1.4
**BODY WEIGHT (kg)**				
Pre-treatment	491 ± 11	567 ± 17	495 ± 10	479 ± 16
Treatment	481 ± 8	565 ± 13	480 ± 7	462 ± 11
Post-treatment	481 ± 12	575 ± 18	480 ± 8	468 ± 17
**RECTAL TEMPERATURE (**°**C)**				
Pre-treatment	38.9 ± 0.2	38.7 ± 0.1	38.8 ± 0.1	39.0 ± 0.2
Treatment	39.1 ± 0.1	39.0 ± 0.1	38.9 ± 0.1	39.2 ± 0.1
Post-treatment	39.0 ± 0.2	38.8 ± 0.2	38.8 ± 0.2	39.0 ± 0.2

Plasma for prolactin analysis were collected daily and analyzed by ELISA. Weights and rectal temperatures were recorded daily. Data analyzed by repeated measures mixed model factorial analysis of variance. Prolactin: Treatment p = 0.554; Experimental Period p < 0.001; Treatment^*^Experimental Period interaction p = 0.4711. Weight: Treatment p = 0.102; Experimental Period p = 0.023; Treatment^*^Experimental Period p = 0.668. Rectal temperature: Treatment p = 0.536; Experimental period p = 0.073; Treatment^*^Experimental Period p = 0.984.

No treatment-specific differences in cow body weight were observed during the study period. There was no Treatment^*^Experimental Period interaction (*p* = 0.668). Weight varied by Experimental Period (*p* = 0.023). Across all treatment groups, cows lost an average of 9.1 kg from the pre-treatment period to the treatment period (*p* = 0.013) and gained an average of 4.5 kg from the treatment period to the post-treatment period (*p* = 0.008).

There was no Treatment (*p* = 0.536) or Treatment^*^Experimental Period interaction for rectal temperature (*p* = 0.984).

### Hemodynamic endpoints

Complete data on hemodynamic variables and parameters from ultrasonographic analyses for each of the three arteries are presented in Supplementary Tables 1, 2.

There was a Treatment^*^Experimental Period interaction for caudal artery diameter (*p* = 0.009), peak systolic velocity (*p* = 0.024), pulsatility index (*p* = 0.007), blood volume per pulse (*p* = 0.001), and blood flow per minute (*p* = 0.007). End diastolic velocity (*p* = 0.002), resistivity index (*p* < 0.001), and pulse rate (*p* = 0.018) of the caudal artery differed for the Experimental Period only (i.e., no Treatment effect or Interaction). For the median sacral artery diameter, there was a Treatment^*^Experimental Period interaction (*p* = 0.011). No treatment effects in any other hemodynamic parameters were recorded for the median sacral artery except for the Experimental Period differences in the peak systolic velocity (*p* = 0.027), end diastolic velocity (*p* = 0.023), pulsatility index (*p* = 0.003), and resistivity index (*p* = 0.009). Pulse rate for the internal iliac artery had a Treatment^*^Experimental Period interaction (*p* = 0.011) and end diastolic velocity varied by Treatment (*p* = 0.024). Other hemodynamic endpoints for the internal iliac artery remained unchanged (i.e., no Treatment effect or Interaction) except for Experimental Period differences in pulsatility index (*p* = 0.02) and resistivity index (*p* = 0.008).

#### Diameter

**Figure 2:**
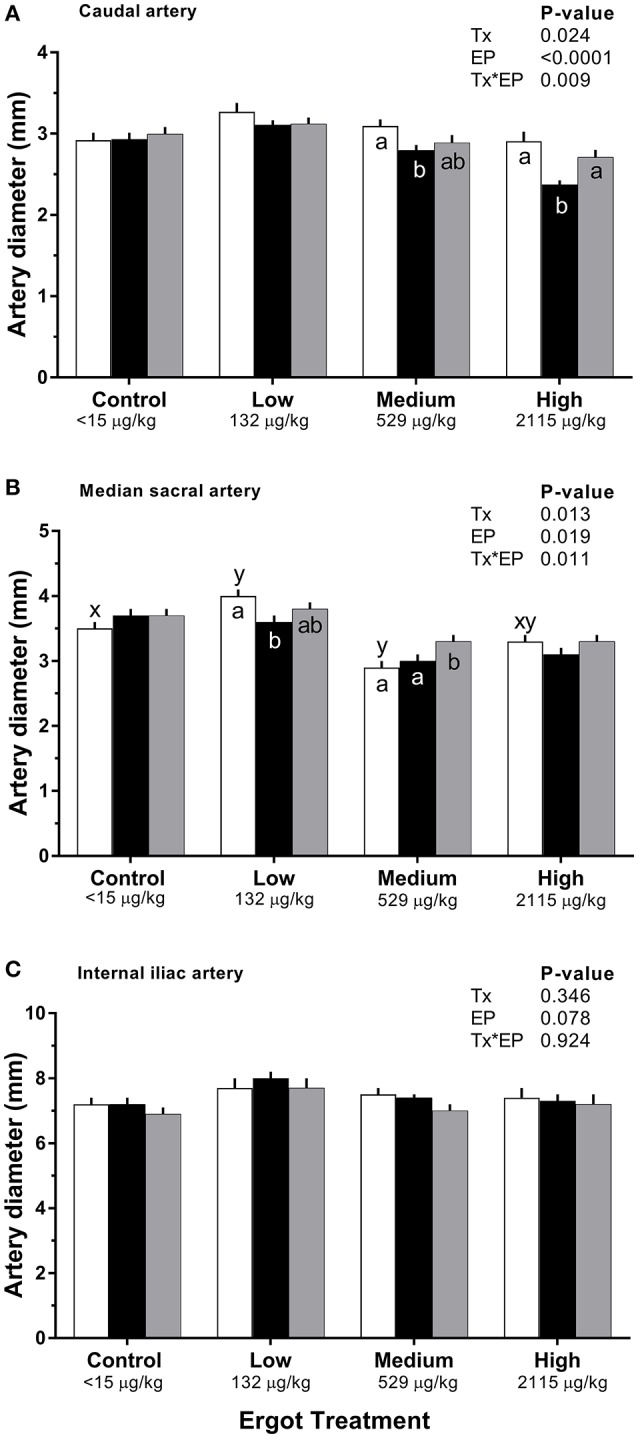
Diameter of the **(A)** caudal artery, **(B)** median sacral artery, and **(C)** internal iliac artery of lactating Hereford cows (*n* = 4 per treatment group) before (4 days), during (7 days), and after (4 days) feeding increasing concentrations of ergot alkaloids in Control (<15 μg/kg), Low (132 μg/kg), Medium (529 μg/kg), and High (2115 μg/kg) ergot groups. Each bar represented the mean±SEM for each experimental period. Repeated measures analysis was used to test for changes in arterial diameter (each artery analyzed individually) for treatment (Tx), experimental period (EP) and their interaction (Tx*EP). Differences among experimental periods within a treatment group (connected bars) are indicated by x and y (*p* < 0.05) and differences among groups during a given treatment period (same colored bars) are indicated by a and b (*p* < 0.05).

Changes in diameter for the three arteries during the 1-week treatment period are given in Figure 2. Each artery was analyzed separately. During the treatment period, caudal artery diameter decreased by 19% (−0.5 mm; *p* = 0.002) in the High ergot treatment compared to the Control group during treatment period (2.9 ± 0.1 mm). Compared to the pre-treatment values, caudal artery diameter during the treatment period decreased by 10% (−0.3 mm; *p* = 0.004) in the Medium ergot group, and by 19% (−0.5 mm; *p* < 0.001) in the High ergot group. No differences (*p* > 0.1) between groups were detected in diameter of the caudal artery during the post-treatment period. Further, the diameter of the caudal artery in the Medium and High groups during the post-treatment period returned to the pre-treatment values (i.e., no difference).

During the treatment period, diameter of the median sacral artery was smaller in the Medium ergot group (−0.7 mm, 19%; *p* = 0.006), and in the High ergot group (−0.6 mm, 17%; *p* = 0.017) compared to the Control group. Pre-treatment diameter of the median sacral artery in the Low ergot group was higher (*p* < 0.042) than the Control, Medium and High ergot groups (+0.5, +1.1, and +0.7 mm, respectively); therefore, inter-group comparisons for the low group were not performed. Within the Low group, the diameter of the median sacral artery during the treatment decrased compared to the pre-treatment value.

For the internal iliac artery diameter, there was no effect of Treatment (*p* = 0.35), Experimental Period (*p* = 0.08), or Treatment^*^Experimental Period (*p* = 0.92).

#### Peak systolic velocity

During the treatment period, there were no differences between the control group and the Medium (*p* = 0.99), or High (*p* = 0.47) ergot treatment groups in PSV of the caudal artery (Supplementary Table 1). Pre-treatment and treatment values did not differ (*P* > 0.22) for PSV value of caudal artery for the Medium and High treatment groups. Caudal artery PSV of the Low ergot group during the treatment and post-treatment period was higher (*p* = 0.015 and 0.001, respectively) than the pre-treatment value.

#### Blood volume per pulse

**Figure 3:**
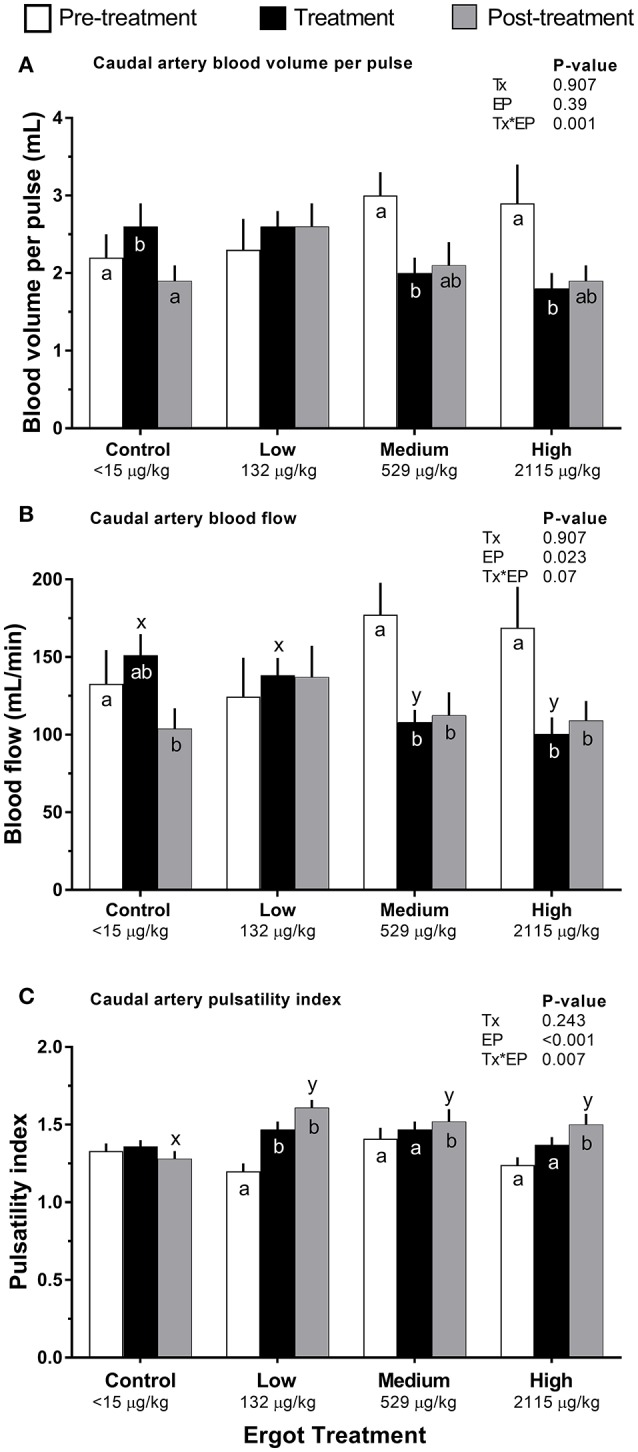
Caudal artery hemodynamic parameters of lactating Hereford cows (*n* = 4 per treatment group) before (4 days, white bars), during (7 days; black bars), and after (4 days, gray bars) feeding increasing concentrations of ergot alkaloids in Control (<15 μg/kg), Low (132 μg/kg), Medium (529 μg/kg), and High (2115 μg/kg) ergot groups. **(A)** pulsatility index (unitless) **(B)** blood volume per pulse (mL), and **(C)** blood flow (mL/min). Data are presented as the mean±SEM for each experimental period. Differences among experimental periods within a treatment group (connected bars) are indicated by x and y (*p* < 0.05) and differences among groups during a given treatment period (same colored bars) are indicated by a and b (*p* < 0.05).

Compared to pre-treatment values, blood volume per pulse of the caudal artery (Figure 3A) decreased in the Medium (−1.0 mL; *p* = 0.004) and High (−1.1 mL; *p* = 0.004) ergot groups during the treatment period. Blood volume per pulse did not differ in these groups when compared to the Control group during the treatment period (*p* > 0.16).

#### Blood flow

Compared to the Control group during the treatment period, blood flow in the caudal artery was reduced by 29% in the Medium treatment (−43 mL/min; *p* = 0.034), and by 34% in the High treatment (−51 mL/min; *p* = 0.021). Caudal artery treatment period and post-treatment blood flow values for the Medium ergot treatment (Figure 3B) were 61% (−69 mL/min; *p* = 0.001) and 63% (−65 mL/min; *p* = 0.007) of the pre-treatment value, respectively. Caudal artery treatment period and post-treatment blood flow values for the High ergot treatment were 59% (−69 mL/min; *p* = 0.006) and 65% (−60 mL/min; *p* = 0.03) of the pre-treatment blood flow. Blood flow was not affected for the median sacral or internal iliac artery (Treatment^*^Experimental Period interaction *p* = 0.175 and 0.366, respectively).

#### Pulsatility index

Compared to the pre-treatment values, caudal artery PI in the Low ergot treatment group (Figure 3C) increased by 23% (*p* = 0.066) and 34% (*p* < 0.001) during the treatment and post-treatment periods, respectively. Post-treatment PI of the caudal artery for ergot treatments was greater than that of the post-treatment PI of the control group. This corresponded to 25% (*p* = 0.002), 18% (*p* = 0.021), and 17% (*p* = 0.033) increases in Low, Medium, and High ergot treatment groups.

#### Pulse rate

In the High ergot treatment, pulse rate measured in the internal iliac artery decreased (*p* = 0.0003) from 62 ± 2 bpm during the pre-treatment to 59 ± 2 bpm during the treatment period (Supplementary Table 1), but both periods did not differ from the corresponding periods in control group (*p* > 0.16). Further, internal iliac arterial pulse rate in the control group during the treatment period was higher (*p* = 0.036) than the post-treatment period (62 ± 1 vs. 60 ± 2 bpm). No difference in pulse rate were detectable when measurements from the caudal artery and median sacral artery were compared.

## Discussion

The present study investigated whether a dose-response relationship existed between total ergot alkaloids in feed and changes in hemodynamic parameters of three arteries in lactating Hereford cows. Although ergot alkaloids are known to suppress plasma prolactin levels (13, 45–47), ergot consumption at 132 (low) 529 (medium) and 2115 (high) μg per kg of dry matter intake per day did not affect plasma prolactin concentrations in the cattle in this study. In addition, body weight and rectal temperature were not affected by ergot treatment. It is noteworthy that the caudal artery diameter decreased in medium (9.7%) and high (19%) ergot groups during treatment period compared to pre-treatment period. Likewise, median sacral artery diameter during the treatment period decreased in the medium and high ergot groups compared to the control group. In contrast, internal iliac artery diameter was not affected. Further, caudal artery pulsatility index increased during post-treatment period for all groups while the volume per beat during the treatment period was lower for medium and high group than the pre-treatment values. Blood flow in the caudal artery during the treatment and post-treatment periods for the medium and high ergot treatments was reduced to < 65% of pre-treatment values. Our results support the notion that peripheral arteries (e.g., the caudal artery) are more sensitive to ergot alkaloids than the more central arteries (e.g., the internal iliac artery).

Ergot alkaloid concentrations at 529 and 2,115 μg/kg of dry matter intake affected the caudal artery hemodynamics following 1 week of daily ergot consumption. Specifically, reduced arterial diameter, blood flow, and blood volume per heart beat were observed. These results are indicative of vasoconstriction and reduced arterial perfusion, both of which are hallmarks of gangrenous ergotism in livestock. Two studies of cattle ingesting endophyte-infected tall fescue detected vasoconstriction in the caudal artery with color Doppler ultrasonography at similar concentrations as in the present study. A decrease in caudal artery cross-sectional area was detectable in beef heifers fed 390 or 790 ergovaline per kg dry matter (25) and those that were fed 850 μg ergovaline per kg recorded a 42% reduction (19). The duration of constriction was dose-dependent wherein the 790 μg concentration of ergot resulted in a longer duration of constriction than 390 μg (25). Likewise, goats exposed to fescue ergovaline and ergovalinine (800 μg/kg of dry matter intake) had reduced cross-sectional areas of the carotid and auricular arteries (48) and horses fed fescue seed containing 4.93 mg/kg ergovaline had reduced blood flow of the palmar artery (49). These previous studies and the present results suggest that concentrations of ergot alkaloids >390 μg/kg cause vasoconstriction in the caudal artery following short-term exposure in feed in beef cows and heifers. It is worth mentioning that ergocristine (48% to total), ergocryptine (17%), and ergotamine (>11%) are major alkaloids in ergot sclerotia in western Canadian grain (50) therefore, direct comparison between *C. purpurea* alkaloids and endophyte alkaloids, and between species should be made with caution due to differing alkaloid profiles and potencies, and species-specific sensitivity to ergot alkaloids.

Our study detected an increased pulsatility index in the low ergot treatment (but not in the medium or high groups) during the treatment period. Further increased PI was recorded during the post-treatment period of all ergot treatments. Our finding indicates an increase in arterial resistance to flow suggesting that some effects of ergot are delayed. Vessel bioassay studies of fescue ergot alkaloids have also noted that there is a persistent effect on the cardiovascular system, despite ergot alkaloid removal [(13, 21, 22, 28, 51, 52)] supporting our notion.

This study is the first to report on the effect of ergot alkaloid exposure on bovine median sacral artery. The median sacral artery is the internal counterpart to the caudal artery, and thus served as a test of arterial location for hemodynamic changes. As seen in the caudal artery, constriction was observed (i.e., reduced diameter) in the medium and high ergot treatments. However, reduction in blood flow was not seen in this artery, suggesting that the caudal artery was more responsive to ergot exposure. The difference in response between the two arteries may be related to the influence of ambient temperature, however this remains to be investigated.

The internal iliac artery appeared to be the least responsive to ergot treatment. There were no changes in hemodynamic parameters that would indicate vasoconstriction. A potential explanation for these results (compared to the caudal and median sacral arteries) could be related to the relative proportion of smooth muscles in their tunica media, size of the vessels, differences in number of bioamine receptors, or variations in receptor sensitivity. Diameter of the internal iliac artery was ~2.5 times larger than that of the caudal artery (Figure 1). The internal iliac artery branches off the aorta (53, 54), thus receives large volumes of blood for delivering to pelvic organs such as the uterus and vagina. The internal iliac artery is more elastic than more peripheral arteries to compensate for arterial pressure fluctuations during systole and diastole. The median sacral artery is also a branch of the aorta, albeit much smaller than the internal iliac and eventually terminates in the caudal artery supplying the tail. The peripheral location of the caudal artery implies that it has higher smooth muscle content and lower elastic content than the other two arteries (55, 56). Since ergot alkaloids act on bioamine receptors in vascular smooth muscle (18), it would be reasonable to assume that the effect of the alkaloids would be more prominent in arteries with higher proportion of smooth muscles in their tunica media. The observation that the caudal artery is most sensitive to ergot reinforces the well-known feature of ergot as a peripheral vasoconstrictor agent. Peripheral appendages, such as the ear tips, tail tips, and hooves are affected by ergotism (13, 57–60).

Reduction in circulating prolactin concentrations in cows exposed to ergot compared to control animals and pre-ergot values were not observed during the present study. Numerous studies indicate that prolactin production is suppressed following ergot alkaloid exposure (45–47, 61–69). Prolactin secretion is affected by multiple environmental and physical factors, including stress, ambient temperature, photoperiod, mechanical stimulation (i.e., calf suckling, handling), and physiological status (i.e., pregnant, lactating) (70, 71). The presence of these factors may increase or decrease the basal prolactin levels in cattle. It is noteworthy that by design of the experiment, we tested the effect of very low amounts, i.e., subclinical levels of ergot consumption. Current Canadian standards permit 2,000–3,000 μg/kg of ergot alkaloids in feed (38). Animals in this study ate between 100 and 2,100 μg/kg of dry matter intake for 7 days. It is likely that plasma prolactin was not affected by the amount of ergot fed in this study. The present study suggests that subclinical vasoactive effects of ergot may be a more sensitive bioindicators of exposure as compared to prolactin alterations. It remains to be examined if prolactin suppression may be affected after chronic exposure whereas cardiovascular alterations may be more rapid in nature. The most sensitive endpoint of ergot exposure in cattle may be influenced by both the dose and duration of exposure, not dose alone.

It should be noted that no clinical signs of gangrenous ergotism were observed throughout the study. Commonly cited symptoms of gangrenous ergotism include bilateral hindlimb lameness, hoof swelling, and, eventually, loss of the tail switch, ear tips, and, in the most severe cases, hoof loss. The authors did not anticipate adverse clinical effects in the ergot-exposed animals in this study, due to both the moderate summer climate in which this study was conducted and the short duration of the exposure. Had this study been conducted during cold ambient temperatures, however, clinical signs may have developed as cold temperatures exacerbate gangrenous symptoms of ergot alkaloid exposure (13, 16). The current Canadian permissible levels of ergot alkaloids in feed do not account for duration of exposure or the season of exposure. Further studies are required to characterize the relationship between ergot alkaloid concentration in feed, ambient temperature, duration of exposure, and development of subclinical and clinical changes in peripheral vasculature.

In conclusion, our hypothesis that of increasing ergot concentrations having a dose-related response on arterial hemodynamic parameters was partially supported. Vasoconstriction and reduced perfusion were observed in the caudal artery of beef cows at 529 and 2115 μg/kg dry matter, but not 132 μg/kg dry matter. Increased resistance to flow was observed in all ergot treatments despite removal of ergot from the feed. Constriction was also seen in the median sacral artery at 529 and 2115 μg/kg dry matter, but perfusion was unaffected. The internal iliac artery was largely unaffected by treatment. Plasma prolactin levels and rectal temperatures were not affected at these low levels of ergot exposure for 7 days. The threshold for vasoconstriction following short term ergot exposure seems to fall between 132 and 529 μg/kg dry matter. Future work should include assessment of arterial responses following long-term ergot alkaloid exposure in feed of cattle.

## Data availability statement

The datasets generated for this study are available upon request.

## Author contributions

VC was responsible for carrying out the research, data analysis, laboratory sample analysis, and manuscript preparation. AN was involved in Doppler ultrasound data acquisition and analysis. JM contributed to ration formulation, nutritional considerations, and feeding. BB was involved in dose-selection, experimental design, and consultation. TG was involved in sample preparation and processing. JS was the principle investigator of the grant and contributed toward concept development, hypotheses formulation, experimental design, establishment of Doppler ultrasound procedures, oversight of the study, statistical analyzes and manuscript preparation/revisions.

### Conflict of interest statement

The authors declare that the research was conducted in the absence of any commercial or financial relationships that could be construed as a potential conflict of interest.
