# Supplementary material for: Arterial Responses to Acute Low-Level Ergot Exposure in Hereford Cows
Source: Front Vet Sci. 2018 Oct 16;5:240. doi: 10.3389/fvets.2018.00240 (PMC6198079; doi:10.3389/fvets.2018.00240)
Supplement: Supplementary file 1 [file Table_1.DOCX]

Supplementary Material

Arterial responses to acute LOW-LEVEL ergot EXPOSURE in Hereford cows

Cowan V^1,2*^, Neumann A^1^, McKinnon J^3^, Blakley B^1,2^, Grusie T^1^, Singh J^1^.

^1^Veterinary Biomedical Sciences, Western College of Veterinary Medicine, University of Saskatchewan, Saskatoon SK Canada

^2^Toxicology Centre, University of Saskatchewan, Saskatoon SK Canada

^3^Animal and Poultry Science, College of Agriculture and Bioresources, University of Saskatchewan, Saskatoon SK Canada

*** Correspondence:** Dr. Jaswant Singh jaswant.singh@usask.ca

# Supplementary Data

Appendix I. Syntax for statistical analysis in SAS Proc mixed.

The model was tested with nine covariance matrices to determine the best fit using the lowest AICC score. Word “simple” in the following syntax (i.e., “type=”) was replaced with “cs”, “csh”, “toep”, “toep(1)”, hf, “ar(1)”, “arh(1)”, and “ante(1)” to run compound symmetry, heterogeneous compound symmetry, Toeplitz, banded Toeplitz, Huynh-Feldt, autoregressive, heterogeneous autoregressive, and ante-dependence covariance matrices, respectively. ID = unique identifier for each of the 16 cows in the study; Group = Control, Low, Medium or High; Day = days of data collection (-4 to 9, 14); Period = Before, During or After; Rep = Replicate 1 or Replicate 2; Diam = Recorded diameter of the blood vessel.

ODS graphics on**;**

**Proc** **mixed** data=WORK.ACUTEERGOT cl covtest;

class ID Group Day Period Rep;

model DIAM = Group|Period / DDFM=kr htype=**3**;

random intercept/subject=Rep;

repeated Day/subject=ID (Group) type=simple;

run;

ODS graphics off**;**

***Once the best fit covariance matrix was selected, the final analysis was run***;

ODS graphics on**;**

**Proc** **mixed** data=WORK.ACUTEERGOT cl covtest plots=residualpanel;

class ID Group Day Period Rep;

model DIAM = Group|Period / DDFM=kr htype=**3**;

random intercept/subject=Rep;

repeated Day/subject=ID (Group) type=cs r rcorr;

lsmeans Group|Period / pdiff;

run;

ODS graphics off**;**

# Supplementary Figures and Tables

| **SUPPLEMENTARY TABLE 1.** Hemodynamic variables (mean ± SEM) of caudal artery, median sacral artery and internal iliac artery measured by Doppler ultrasonography in beef cows (n=4 per treatment group) during the pre-treatment (4 days), treatment (7 days), and post-treatment (4 days) experimental periods to increasing concentrations of ergot alkaloids in their feed in Control, Low, Medium and High groups. Arteries were imaged daily and data were compared by repeated measures mixed procedure. Pair-wise comparisons were performed if the ANOVA p-value was ≤0.05 for the treatment (control, low, medium and high), experimental period (pre-treatment, treatment, post-treatment) or treatment*experimental period interaction term. Superscripts ab indicate differences in columns (among periods within a treatment) and superscripts xy indicate differences in rows (among treatments for a given period). Values with uncommon alphabets are different at p≤0.05 | | | | |
| --- | --- | --- | --- | --- |
|  | Ergot Treatment Group | | | |
| Variable | Control | Low  (132 μg/kg DM) | Medium (529 μg/kg DM) | High (2115 μg/kg DM) |
|  |  |  |  |  |
| Caudal artery |  |  |  |  |
| Diameter (mm) |  |  |  |  |
| Pre-treatment | 2.9 ± 0.1 | 3.3 ± 0.1 | 3.1 ± 0.1^a^ | 2.9 ± 0.1^a^ |
| Treatment | 2.9 ± 0.1^x^ | 3.1 ± 0.1^x^ | 2.8 ± 0.1^bx^ | 2.4 ± 0.1^by^ |
| Post-treatment | 3.0 ± 0.1 | 3.1 ± 0.1 | 2.9 ± 0.1^ab^ | 2.7 ± 0.1^a^ |
| Peak systolic velocity (m/s) |  |  |  |  |
| Pre-treatment | 0.68 ± 0.03 | 0.55 ± 0.03^a^ | 0.76 ± 0.05 | 0.75 ± 0.05 |
| Treatment | 0.71 ± 0.03 | 0.67 ± 0.02^b^ | 0.71 ± 0.03 | 0.75 ± 0.04 |
| Post-treatment | 0.64 ± 0.03 | 0.74 ± 0.05^b^ | 0.71 ± 0.04 | 0.74 ± 0.04 |
| Mean arterial velocity (m/s) |  |  |  |  |
| Pre-treatment | 0.40 ± 0.02 | 0.36 ± 0.02 | 0.43± 0.03 | 0.45 ± 0.03 |
| Treatment | 0.41 ± 0.02 | 0.37 ± 0.01 | 0.40 ± 0.01 | 0.43 ± 0.02 |
| Post-treatment | 0.40 ± 0.02 | 0.39 ± 0.03 | 0.39 ± 0.02 | 0.42 ± 0.02 |
| End diastolic velocity (m/s) |  |  |  |  |
| Pre-treatment | 0.14 ± 0.02 | 0.13 ± 0.02 | 0.15 ± 0.02 | 0.20 ± 0.03 |
| Treatment | 0.15 ± 0.01 | 0.12 ± 0.01 | 0.12 ± 0.01 | 0.18 ± 0.01 |
| Post-treatment | 0.13 ± 0.01 | 0.12 ± 0.02 | 0.12 ± 0.01 | 0.12 ± 0.02 |
| Pulse rate (bpm) |  |  |  |  |
| Pre-treatment | 59 ± 2 | 55 ± 1 | 59 ± 2 | 62 ± 2 |
| Treatment | 58 ± 1 | 52 ± 1 | 56 ± 1 | 58 ± 2 |
| Post-treatment | 56 ± 2 | 52 ± 2 | 55 ± 2 | 59 ± 2 |
|  |  |  |  |  |
| Median sacral artery |  |  |  |  |
| Diameter (mm) |  |  |  |  |
| Pre-treatment | 3.5 ± 0.1^x^ | 4.0 ± 0.1^ay^ | 2.9 ± 0.1^ay^ | 3.3 ± 0.1^xy^ |
| Treatment | 3.7 ± 0.1^x^ | 3.6 ± 0.1^bx^ | 3.0 ± 0.1^ay^ | 3.1 ± 0.1^y^ |
| Post-treatment | 3.7 ± 0.1 | 3.8 ± 0.1^ab^ | 3.3 ± 0.1^b^ | 3.3 ± 0.1 |
| Peak systolic velocity (m/s) |  |  |  |  |
| Pre-treatment | 1.16 ± 0.06 | 1.24 ± 0.10 | 1.25 ± 0.06 | 1.17 ± 0.06 |
| Treatment | 1.30 ± 0.05 | 1.33 ± 0.05 | 1.28 ± 0.05 | 1.32 ± 0.07 |
| Post-treatment | 1.22 ± 0.08 | 1.28 ± 0.07 | 1.28 ± 0.09 | 1.40 ± 0.08 |
| Mean arterial velocity (m/s) |  |  |  |  |
| Pre-treatment | 0.61 ± 0.03 | 0.59 ± 0.05 | 0.64 ± 0.04 | 0.55 ± 0.02 |
| Treatment | 0.63 ± 0.02 | 0.62 ± 0.03 | 0.62 ± 0.02 | 0.63 ± 0.03 |
| Post-treatment | 0.60 ± 0.04 | 0.59 ± 0.03 | 0.60 ± 0.04 | 0.63 ± 0.03 |
| End diastolic velocity (m/s) |  |  |  |  |
| Pre-treatment | 0.25 ± 0.02 | 0.25 ± 0.03 | 0.26 ± 0.02 | 0.26 ± 0.02 |
| Treatment | 0.25 ± 0.02 | 0.24 ± 0.02 | 0.20 ± 0.01 | 0.31 ± 0.02 |
| Post-treatment | 0.24 ± 0.03 | 0.23 ± 0.02 | 0.21 ± 0.01 | 0.28 ± 0.03 |
| Pulse rate (bpm) |  |  |  |  |
| Pre-treatment | 62 ± 3 | 54 ± 1 | 60 ± 2 | 63 ± 2 |
| Treatment | 59 ± 1 | 53 ± 1 | 58 ± 2 | 59 ± 2 |
| Post-treatment | 58 ± 2 | 53 ± 2 | 57 ± 2 | 58 ± 3 |
|  |  |  |  |  |
| Internal iliac artery |  |  |  |  |
| Diameter (mm) |  |  |  |  |
| Pre-treatment | 7.2 ± 0.2 | 7.7 ± 0.3 | 7.5 ± 0.2 | 7.4 ± 0.3 |
| Treatment | 7.2 ± 0.2 | 8.0 ± 0.2 | 7.4 ± 0.1 | 7.3 ± 0.2 |
| Post-treatment | 6.9 ± 0.2 | 7.7 ± 0.3 | 7.0 ± 0.2 | 7.2 ± 0.3 |
| Peak systolic velocity (m/s) |  |  |  |  |
| Pre-treatment | 0.97 ± 0.06 | 0.88 ± 0.05 | 0.94 ± 0.04 | 0.98 ± 0.05 |
| Treatment | 0.93 ± 0.04 | 0.94 ± 0.03 | 0.95 ± 0.03 | 0.99 ± 0.04 |
| Post-treatment | 0.98 ± 0.05 | 1.07 ± 0.06 | 0.95 ± 0.04 | 1.04 ± 0.07 |
| Mean arterial velocity (m/s) |  |  |  |  |
| Pre-treatment | 0.50 ± 0.03 | 0.43 ± 0.02 | 0.47 ± 0.02 | 0.47 ± 0.02 |
| Treatment | 0.49 ± 0.02 | 0.45 ± 0.01 | 0.51 ± 0.02 | 0.49 ± 0.02 |
| Post-treatment | 0.49 ± 0.02 | 0.50 ± 0.02 | 0.47 ± 0.02 | 0.51 ± 0.03 |
| End diastolic velocity (m/s) |  |  |  |  |
| Pre-treatment | 0.20 ± 0.01 | 0.21 ± 0.01 | 0.19 ± 0.01 | 0.22 ± 0.01 |
| Treatment | 0.19 ± 0.005 | 0.19 ± 0.01 | 0.19 ± 0.01 | 0.22 ± 0.01 |
| Post-treatment | 0.19 ± 0.01 | 0.19 ± 0.01 | 0.19 ± 0.01 | 0.22 ± 0.01 |
| Pulse rate (bpm) |  |  |  |  |
| Pre-treatment | 61 ± 2^ab^ | 55 ± 2 | 60 ± 2 | 62 ± 2^a^ |
| Treatment | 62 ± 1^a^ | 54 ± 2 | 59 ± 2 | 59 ± 2^b^ |
| Post-treatment | 60 ± 2^b^ | 55 ± 2 | 57 ± 2 | 60 ± 2^ab^ |

| **SUPPLEMENTARY TABLE 2**. Calculated hemodynamic parameters (mean ± SEM) of caudal artery, median sacral artery and internal iliac artery measured by Doppler ultrasonography in beef cows (n=4 per treatment group) during the pre-treatment (4 days), treatment (7 days), and post-treatment (4 days) experimental periods to increasing concentrations of ergot alkaloids in their feed in Control, Low, Medium and High groups. Arteries were imaged daily and data were compared by repeated measures mixed procedure. Pair-wise comparisons were performed if the ANOVA p-value was ≤0.05 for the treatment (control, low, medium and high), period (pre-treatment, treatment, post-treatment) or treatment*period interaction term. Superscripts ab indicate differences in columns (among periods within a treatment) and superscripts xy indicate differences in rows (among treatments for a given period). Values with uncommon alphabets are different at p≤0.05ß | | | | |
| --- | --- | --- | --- | --- |
|  | Ergot Treatment Group | | | |
|  | Control | Low  (132 μg/kg DM) | Medium (529 μg/kg DM) | High (2115 μg/kg DM) |
|  |  |  |  |  |
| Caudal artery |  |  |  |  |
| Blood volume per pulse (mL) |  |  |  |  |
| Pre-treatment | 2.2 ± 0.3^a^ | 2.3 ± 0.4 | 3.0 ± 0.3^a^ | 2.9 ± 0.5^a^ |
| Treatment | 2.6 ± 0.3^b^ | 2.6 ± 0.2 | 2.0 ± 0.2^b^ | 1.8 ± 0.2^b^ |
| Post-treatment | 1.9 ± 0.2^a^ | 2.6 ± 0.3 | 2.1 ± 0.3^ab^ | 1.9 ± 0.2^ab^ |
| Blood flow (mL/min) |  |  |  |  |
| Pre-treatment | 133 ± 22^ab^ | 125 ± 25 | 177 ± 20^a^ | 169 ± 26^a^ |
| Treatment | 151 ± 14^bx^ | 138 ± 11^x^ | 108 ± 8^by^ | 100 ± 11^by^ |
| Post-treatment | 104 ± 13^a^ | 137 ± 20 | 112 ± 15^b^ | 109 ± 13^b^ |
| Pulsatility index |  |  |  |  |
| Pre-treatment | 1.33 ± 0.05 | 1.20 ± 0.05 | 1.41 ± 0.07 | 1.24 ± 0.05^a^ |
| Treatment | 1.36 ± 0.04 | 1.47 ± 0.05 | 1.47 ± 0.05 | 1.37 ± 0.05^ab^ |
| Post-treatment | 1.28 ± 0.05^x^ | 1.61 ± 0.05^y^ | 1.52 ± 0.08^y^ | 1.50 ± 0.07^by^ |
| Resistivity index |  |  |  |  |
| Pre-treatment | 0.78 ± 0.02 | 0.77 ± 0.01 | 0.77 ± 0.02 | 0.74 ± 0.02 |
| Treatment | 0.78 ± 0.01 | 0.82 ± 0.01 | 0.83 ± 0.01 | 0.77 ± 0.01 |
| Post-treatment | 0.79 ± 0.01 | 0.84 ± 0.01 | 0.83 ± 0.01 | 0.83 ± 0.02 |
|  |  |  |  |  |
| Median sacral artery |  |  |  |  |
| Blood volume per pulse (mL) |  |  |  |  |
| Pre-treatment | 6.1 ± 0.6 | 8.5 ± 0.9 | 4.3 ± 0.3 | 4.5 ± 0.4 |
| Treatment | 7.1 ± 0.5 | 7.5 ± 0.6 | 4.7 ± 0.3 | 5.3 ± 0.5 |
| Post-treatment | 6.7 ± 0.6 | 7.4 ± 0.6 | 5.9 ± 0.8 | 6.0 ± 0.7 |
| Blood flow (mL/min) |  |  |  |  |
| Pre-treatment | 364 ± 35 | 471 ± 52 | 259 ± 20 | 282 ± 23 |
| Treatment | 408 ± 24 | 404 ± 38 | 264 ± 18 | 296 ± 21 |
| Post-treatment | 387 ± 30 | 402 ± 39 | 325 ± 38 | 340 ± 36 |
| Pulsatility index |  |  |  |  |
| Pre-treatment | 1.51 ± 0.05 | 1.66 ± 0.08 | 1.61 ± 0.07 | 1.66 ± 0.11 |
| Treatment | 1.71 ± 0.06 | 1.80 ± 0.06 | 1.75 ± 0.05 | 1.61 ± 0.07 |
| Post-treatment | 1.69 ± 0.08 | 1.80 ± 0.10 | 1.78 ± 0.07 | 1.79 ± 0.11 |
| Resistivity index |  |  |  |  |
| Pre-treatment | 0.78 ± 0.01 | 0.80 ± 0.02 | 0.79 ± 0.02 | 0.76 ± 0.02 |
| Treatment | 0.80 ± 0.01 | 0.82 ± 0.01 | 0.84 ± 0.01 | 0.76 ± 0.02 |
| Post-treatment | 0.80 ± 0.02 | 0.81 ± 0.02 | 0.83 ± 0.01 | 0.80 ± 0.02 |
|  |  |  |  |  |
| Internal iliac artery |  |  |  |  |
| Blood volume per pulse (mL) |  |  |  |  |
| Pre-treatment | 21.2 ± 2.4 | 22.3 ± 1.8 | 21.3 ± 1.4 | 20.2 ± 1.6 |
| Treatment | 20.5 ± 1.6 | 25.8 ± 1.3 | 22.7 ± 1.0 | 21.7 ± 1.4 |
| Post-treatment | 19.5 ± 1.9 | 25.6 ± 1.9 | 19.5 ± 1.2 | 21.8 ± 1.9 |
| Blood flow (mL/min) |  |  |  |  |
| Pre-treatment | 1254 ± 116 | 1214 ± 94 | 1273 ± 91 | 1242 ± 86 |
| Treatment | 1240 ± 81 | 1365 ± 54 | 1310 ± 53 | 1256 ± 86 |
| Post-treatment | 1125 ± 86 | 1398 ± 103 | 1108 ± 71 | 1292 ± 130 |
| Pulsatility index |  |  |  |  |
| Pre-treatment | 1.58 ± 0.10 | 1.56 ± 0.06 | 1.58 ± 0.04 | 1.61 ± 0.05 |
| Treatment | 1.50 ± 0.05 | 1.68 ± 0.05 | 1.53 ± 0.04 | 1.59 ± 0.04 |
| Post-treatment | 1.66 ± 0.08 | 1.79 ± 0.08 | 1.65 ± 0.07 | 1.62 ± 0.08 |
| Resistivity index |  |  |  |  |
| Pre-treatment | 0.79 ± 0.01 | 0.76 ± 0.01 | 0.79 ± 0.01 | 0.77 ± 0.01 |
| Treatment | 0.78 ± 0.01 | 0.80 ± 0.01 | 0.80 ± 0.01 | 0.77 ± 0.01 |
| Post-treatment | 0.80 ± 0.01 | 0.82 ± 0.01 | 0.80 ± 0.01 | 0.78 ± 0.02 |
|  |  |  |  |  |
